# Increased Herpes simplex virus 1, Toxoplasma gondii and Cytomegalovirus antibody concentrations in severe mental illness

**DOI:** 10.1038/s41398-024-03198-y

**Published:** 2024-12-18

**Authors:** Dimitrios Andreou, Nils Eiel Steen, Kjetil Nordbø Jørgensen, Thor Ueland, Laura A. Wortinger, Lynn Mørch-Johnsen, Ina Drabløs, Tereza Calkova, Robert H. Yolken, Ole A. Andreassen, Ingrid Agartz

**Affiliations:** 1 Department of Psychiatric Research, Diakonhjemmet Hospital, Oslo, Norway; 2 Norwegian Centre for Mental Disorders Research (NORMENT), Institute of Clinical Medicine, University of Oslo, Oslo, Norway; 3 Centre for Psychiatry Research, Department of Clinical Neuroscience, Karolinska Institutet & Stockholm Health Care Services, Stockholm Region, Stockholm, Sweden; 4 Norwegian Centre for Mental Disorders Research (NORMENT), Division of Mental Health and Addiction, Oslo University Hospital, Oslo, Norway; 5 Division of Mental Health and Addiction, Vestre Viken Hospital Trust, Drammen, Norway; 6 Research Institute of Internal Medicine, Oslo University Hospital, Oslo, Norway; 7 Institute of Clinical Medicine, University of Oslo, Oslo, Norway; 8 Thrombosis Research Center (TREC), Division of internal medicine, University hospital of North Norway, Tromsø, Norway; 9 Department of Psychiatry & Department of Clinical Research, Østfold Hospital, Grålum, Norway; 10 Region Vastmanland – Uppsala University, Centre for Clinical Research, Vastmanland Hospital Vasteras, Västerås, Sweden; 11 Stanley Division of Developmental Neurovirology, Department of Pediatrics, Johns Hopkins University School of Medicine, Baltimore, MD US

**Keywords:** Schizophrenia, Bipolar disorder

## Abstract

Infections with *Cytomegalovirus* (CMV), *Herpes simplex virus 1* (HSV1) and *Toxoplasma gondii* (TG) have been implicated in severe mental illness. All three pathogens have high seroprevalence in the human population, are neurotropic and establish a persistent infection. We hypothesized that exposed (seropositive) patients with severe mental illness would show higher immunoglobulin G (IgG) concentrations than exposed healthy controls (HC). We included 765 patients with severe mental illness (schizophrenia n = 515, bipolar disorder n = 250) and 541 HC. CMV, HSV1 and TG IgG seropositivity and concentrations were measured with immunoassays (seropositivity: CMV, n = 447 patients vs. 296 HC; HSV1, n = 355 vs. 238; and TG, n = 159 vs. 126). Among seropositive participants, patients had higher HSV1 (p < 0.001) and TG (p = 0.003) IgG concentrations than HC. Stratifying by diagnosis, both schizophrenia (p = 0.001) and bipolar disorder (p = 0.001) had higher HSV1 IgG concentrations, while schizophrenia only had higher TG (p = 0.009) and CMV (p = 0.045) IgG concentrations than HC. In SZ, higher HSV1 IgG concentrations were associated with higher psychotic (p = 0.030) and manic (p = 0.008) symptom scores, but only among CMV- or TG-infected patients which suggests synergistic effects. Among all participants, HSV1 IgG concentrations were inversely associated with interleukin-18 (p < 0.001) and positively associated with high-sensitivity C-reactive protein (p = 0.002) and B cell-activating factor (p = 0.004), possibly indicating T cell exhaustion, enhanced inflammation, and increased B-cell response, respectively. Patients with severe mental illness exhibit a heightened immune system response to HSV1, TG, and CMV infections suggesting immune system dysfunction and/or a more severe infection. For HSV1, higher IgG concentrations were linked to a greater clinical burden.

## Introduction

Schizophrenia (SZ) and bipolar disorder (BP) each affect approximately 1% of the global population [1, 2]. Both disorders constitute severe mental illnesses (SMI) characterized by complex and partially unknown etiological underpinnings wherein both genetic and environmental factors are implicated [1, 2]. Previous research has implicated the neurotropic pathogens *Cytomegalovirus* (CMV), *Herpes simplex virus 1* (HSV1), and *Toxoplasma gondii* (TG) in SMI, albeit with varying degrees of consistency between findings [3–7]. It is noteworthy that all three agents exhibit a high universal seroprevalence and are neurotropic; the primary infection within immunocompetent hosts is typically either asymptomatic or oligosymptomatic, but the pathogens typically establish life-long latency with periodic reactivation phenomena [3, 8–11].

Two pivotal avenues of research merit particular attention: First, a higher frequency of seropositive individuals in patient groups compared to healthy controls (HC) could suggest a role of the pathogens in the risk of developing SZ or BP. However, during the past decades, studies on the seroprevalence of these pathogens in SZ and BP have yielded conflicting results, which renders new studies of larger and better-characterized samples imperative. More recent large-scale studies reveal that TG seropositivity may be associated with SZ [4], particularly in patients with a recent onset of psychosis [12]. Recent meta-analyses have linked TG seropositivity to both SZ and BP [7] but failed to link CMV seropositivity to SZ [5]. Interestingly, in a study of a large cohort of Danish blood donors, CMV seropositivity was associated with mood disorders and the presence of any psychiatric disorder [4]. In a previous study of a smaller sample largely overlapping with the current sample, we did not find any difference in CMV antibody positivity in patients with SZ or BP relative to HC (antibody positivity 56%, 54% and 55%, respectively) [13].

Second, independent of the prevalence of seropositivity in SMI, patients may be, when contracting the pathogens, susceptible to their deleterious ramifications, whereas HC may be less or not susceptible. The hypothesized increased susceptibility in patients could be due to aberrations within the immune system. There is compelling evidence that SMI, particularly SZ, is linked to immune system abnormalities, encompassing both innate and adaptive immune responses [14–17]. This immune dysregulation can result in a less efficient initial immune response and inadequate control of pathogen replication as well as more frequent reactivations later in the course of the illness, resulting in higher viral/parasitic loads. This may in turn lead to an enhanced or prolonged antibody response. Further, SMI patients have been shown to demonstrate B-cell hyperactivity [18], with the hyperactivated cells producing antibodies against the pathogens as well as interleukins and autoantibodies independent of infection severity. In sum, we hypothesize that after exposure to neurotropic pathogens, SMI patients, as compared to HC, will show a heightened host immune system response, expressed with increased antibody concentrations, which may indicate immune system dysfunction and/or more severe infection in SMI.

We here aimed to examine circulatory CMV, HSV1, and TG immunoglobulin G (IgG) positivity ( = seropositivity) and IgG concentrations in an adult sample of SZ, BP, and HC. We hypothesized higher IgG concentrations in seropositive patients compared to seropositive HC, and among seropositive patients, positive associations between IgG levels and symptom severity. Further, in line with our previous results on CMV, we hypothesized a similar frequency of IgG seropositivity in patient groups and HC [13]. Finally, we hypothesized associations of all three IgG levels with selected immune markers irrespective of diagnosis, in particular, with elevated interleukin 18 (IL-18), high-sensitivity C-reactive protein (hs-CRP), B cell-activating factor (BAFF) and BAFF/A proliferation-inducing ligand (APRIL) ratio; this may support the notion that the measured IgG levels are linked to enhanced inflammation, increased B-cell function response and central nervous system pathology [19–21].

## Methods and material

### Participants

We selected participants with available CMV, HSV1, and TG data from the Thematically Organized Psychosis (TOP) research study (2003–2017). The TOP study is part of the Norwegian Centre for Mental Disorders Research (NORMENT, Oslo, Norway, including participants in the age range 18–65 years; www.med.uio.no/norment/english). We recruited the participating patients (outpatients or inpatients) from psychiatric units in Oslo, Norway, and the HC randomly from the same catchment area using the national population register of Norway. We assessed the patients with the Structured Clinical Interview (SCID-I) for the Diagnostic and Statistical Manual of Mental Disorders, fourth edition (DSM-IV) [22], and included those with SZ or BP spectrum disorders. We screened the HC with the Primary Care Evaluation of Mental Disorders (Prime-MD) [23], and excluded those with previous or current psychiatric disorders including substance use disorder (including alcohol use disorder) or first-degree relatives with SMI. We excluded individuals with previous moderate or severe head injury, neurological disorders, or medical conditions that could affect brain function.

The current sample consisted of 1306 participants: 765 patients with SMI, i.e., 515 patients with SZ spectrum disorders (SZ (n = 295), schizophreniform disorder (n = 31), schizoaffective disorder (n = 74), delusional disorder (n = 34), brief psychotic disorder (n = 5) and psychotic disorder not otherwise specified (n = 76)), and 250 patients with BP spectrum disorders (BP I (n = 169), BP II (n = 69) and BP not otherwise specified (n = 12)), and 541 HC.

The authors assert that all procedures contributing to this work comply with the ethical standards of the relevant national and institutional committee on human research and with the Helsinki Declaration. The study was approved by the Regional Committee for Medical Research Ethics South East Norway and the Norwegian Data Inspectorate. All participants gave written informed consent.

### Measures and medication

Education level is a socioeconomic status indicator capturing the shift from parental to own socioeconomic status [24]. We have here used education years as a proxy indicator for socioeconomic status for both patients and HC. For all participants we assessed handedness (right-handedness vs. left-handedness/ambidexterity), alcohol use with the Alcohol Use Disorder Identification Test (AUDIT) [25], and drug use with the Drug Use Disorder Identification Test (DUDIT) [26]. Further, we assessed full scale current intelligence quotient (IQ) with a licensed translated version of the Wechsler Abbreviated Scale of Intelligence (WASI) [27]. We evaluated the patients with the Positive and Negative Syndrome Scale (PANSS) [28], the Young Mania Rating Scale (YMRS) [29], and the Inventory of Depressive Symptoms, clinician-rated (IDS-C) [30]. Duration of illness (DOI) was defined as the time passed since the first psychotic episode and the first affective episode for SZ and BP patients, respectively. We obtained information on current use of antipsychotics, antiepileptics, antidepressants, and lithium by clinical interviews and hospital records, and for patients on antipsychotics, we calculated the current chlorpromazine equivalent doses (CPZ) in mg/day [31].

### Antibody, IL-18, BAFF, APRIL and hs-CRP assessments

Blood samples were drawn from all participants, and serology assessment was performed at the Stanley Neurovirology Laboratory, Johns Hopkins University School of Medicine, Baltimore, MD, USA. CMV, HSV1, and TG IgG concentrations were measured by solid-phase immunoassay techniques and were expressed as continuous (antibody concentrations) and dichotomous measures (seropositivity vs. seronegativity). Methods for antibody measurement and the establishment of cut-offs for antibody positivity based on standards run with each sample have been previously described [32–35]. In short, ratio values for antibody measurements are quantitative with some ceiling effects. They are calculated by dividing the signal generated from the sample by that generated from a standard sample. Plasma levels of IL-18, BAFF, and APRIL (pg/ml) were analysed in duplicate using commercially available antibodies in a 384 format using a combination of a SELMA (Jena, Germany) pipetting robot and a BioTek dispenser/washer, as previously described [36, 37]. Plasma levels of hs-CRP (mg/L) were measured by a particle enhanced immunoturbidimetric method on a Cobas 8000 instrument at the Department of Medical Biochemistry, Oslo University Hospital, (Roche Diagnostics, Basel Switzerland). All samples were tested under code with the laboratory unaware of the demographic or clinical status of the participants.

### Statistics

#### Seropositivity analysis

First, we compared the HSV1, TG, and CMV IgG seropositivity frequencies in patients with SZ, patients with BP, and HC applying chi-square tests. Next, we calculated the pathogen load as the number of seropositivities/infections (0, 1, 2, or 3) and compared the pathogen load frequencies in SZ, BP, and HC applying a chi-square test. Finally, we ran a binary logistic regression model on the SMI/HC status where all three seropositivity variables were inserted in the same model.

#### Main analysis

Next, in bivariate analysis, we assessed group differences between HSV1 seropositive (HSV1+) patients with SMI and HSV1+ HC (applying chi-square tests for categorical variables and t-tests for quantitative variables) in sex, age, education years, handedness, AUDIT and DUDIT as well as the correlation of each variable with HSV1 concentrations (applying point-biserial correlations for binary variables and Spearman’s correlations for quantitative variables). For HSV1+ patients, we also assessed the correlations of DOI, PANSS, YMRS, and IDS-R scores, the current use of antipsychotics, antidepressants, antiepileptic, and lithium, and the CPZ, with HSV1 concentrations (applying point-biserial correlations for binary variables and Spearman’s correlations for quantitative variables). Finally, as HSV1 IgG concentrations were correlated with symptom scores (which is thoroughly described in the results section), we also searched for possible synergistic/antagonistic effects taking account of the CMV and TG IgG status.

HSV1 IgG concentration distribution was highly positively skewed and a logarithmic transformation was applied (log_10_HSV1). In the analysis of covariance (ANCOVA) among HSV1+ participants, we investigated the main effect of patient/control status on log_10_HSV1 controlling for sex, age, and covariates that were significantly correlated with HSV1 in the bivariate analysis.

For TG and CMV, we followed the same steps as for HSV1. TG and CMV were also highly positively skewed, and as for HSV1, we ran ANCOVAs on log_10_TG and log_10_CMV. As we ran three ANCOVAs, we accepted a Bonferroni-corrected alpha level of 0.05/3 = 0.017. Similarly, as we ran three analyses for pathogen-PANSS total score correlations, we accepted a Bonferroni-corrected alpha level of 0.05/3 = 0.017.

Finally, in the whole sample of seropositive patients and HC, we searched for correlations between HSV1, TG, and CMV IgG concentrations and IL-18, hs-CRP, BAFF, and BAFF/APRIL ratio.

#### Post-hoc analysis

We separated the patients into SZ and BP and reran the ANCOVAs with pairwise comparisons (Bonferroni-adjusted). As sensitivity analysis, we ran median regressions on HSV1, TG, and CMV IgG concentrations to confirm the ANCOVA results. Median regression is a special case of the quantile regression and permits adjustment for covariates [38].

All tests were two-sided. We conducted all the analyses with IBM SPSS Statistics 28.

## Results

### Seropositivity analysis

There were no significant differences in the frequency of CMV, HSV1, or TG seropositivity between SZ, BP, and HC (Table 1). There was no difference in pathogen load frequencies between SZ, BP, and HC (Table 1). In the binary logistic regression model, none of the seropositivity variables were associated with the SMI/HC status (b = 0.143, p = 0.213 for CMV, b = 0.078, p = 0.499 for HSV1 and b = −0.156, p = 0.251 for TG).

**Table 1:** Frequencies of Cytomegalovirus (CMV), Herpes simplex virus 1 (HSV1), and Toxoplasma gondii (TG) immunoglobulin G seropositive (CMV+, HSV1+, and TG+, respectively) patients with schizophrenia (SZ), bipolar disorder (BP) and healthy controls (HC).

	SZ N = 515	BP N = 250	HC N = 541	P-values^a^
CMV+ (%)	59.2	56.8	54.7	0.335
HSV1+ (%)	46.8	45.6	44	0.657
TG+ (%)	20.2	22	23.3	0.475
Pathogen load				0.914
0 (%)	21.9	22.8	22.9	
1 (%)	36.9	38.4	38.8	
2 (%)	34.2	30.4	31.6	
3 (%)	7	8.4	6.7	

^a^Chi-square tests.

Pathogen load (number of infections/seropositivities) frequencies by diagnosis are also shown.

### Main analysis

#### Herpes simplex virus 1

In the bivariate analysis of HSV1+ patients with SMI and HSV1+ HC (Table 2), there were no significant differences between SMI and HC in sex distribution, age, or handedness. The SMI group had fewer education years, and higher AUDIT and DUDIT scores than HC (p < 0.001 for all three associations). HSV1 IgG concentrations were inversely correlated with education years, assessed with Spearman’s correlation, r_s_ = −0.186, p < 0.001. Among HSV1+ patients with SMI, HSV1 IgG concentrations were positively correlated with IDS-C score (r_s_ = 0.148, p = 0.016) as well as PANSS total score (r_s_ = 0.135, p = 0.012) (Table 2). Follow-up analysis showed that HSV1 IgG concentrations were positively correlated with both general (r_s_ = 0.127, p = 0.017), positive (r_s_ = 0.117, p = 0.028), and negative (r_s_ = 0.110, p = 0.041) psychotic symptom scores. Among HSV1+ SMI patients as well as among HSV1 + HC, there were no correlations between HSV1 IgG concentrations and IQ (r_s_ = −0.049, p = 0.414, and r_s_ = 0.048, p = 0.465, respectively).

**Table 2:** Group differences between Herpes simplex virus 1 (HSV1) immunoglobulin G (IgG) seropositive (HSV1+) patients with severe mental illness and healthy controls (HC) in sex, age, education years, handedness (right-handedness vs. left-handedness/ambidexterity), alcohol use disorder identification test (AUDIT) and drug use disorder identification test (DUDIT) scores.

	HSV1+ patients		HSV1+ HC		P-value^b^	Correlation with HSV1	
	N^a^	Mean (SD) or %	N^a^	Mean (SD) or %		±	P-value^c^
Sex (% women)	355	45.4	238	43.7	0.691	+	0.263
Age (years)	355	33.4 (10.8)	238	34.7 (8.7)	0.092	-	0.573
Education years	321	12.5 (2.5)	238	14.3 (2.2)	**<0.001**	-	**<0.001**
Handedness (% right-handedness)	321	89.4	238	89.5	0.973	+	0.158
AUDIT score	257	7.4 (7.1)	133	5.7 (3.4)	**<0.001**	-	0.263
DUDIT score	281	3.5 (7.5)	139	0.4 (1.7)	**<0.001**	+	0.270
DOI (years)	347	10 (9.4)				-	0.153
PANSS total score	346	56.9 (16.7)				+	**0.012**
YMRS score	301	4.6 (4.9)				+	0.118
IDS-C	263	17.7 (12)				+	**0.016**
On antipsychotics (%)	355	76.3				-	0.685
CPZ (mg/day)	266	327.6 (273.3)				-	0.694
On antidepressants (%)	355	31				+	0.585
On antiepileptics (%)	355	25.1				-	0.787
On lithium (%)	355	7.3				-	0.685

For patients, the duration of illness (DOI), Positive and Negative Syndrome Scale (PANSS) score, Young Mania Rating Scale (YMRS) score, Inventory of Depressive Symptoms, clinician rated, score (IDS-C), the percentage of patients on antipsychotics, antidepressants, antiepileptics, and lithium as well as the chlorpromazine equivalent doses (CPZ) among patients on antipsychotics are also shown. P values < 0.05 shown in bold.

^a^Number of participants with data in each variable.

^b^Chi-square test or t-test (Welch’s t-test in case of unequal variance).

^c^Point-biserial correlations for binary variables; Spearman’s correlations for quantitative variables.

Stratifying by diagnosis, among HSV1+ patients with SZ, HSV1 IgG concentrations were positively correlated with total (r_s_ = 0.142, p = 0.030), general (r_s_ = 0.132, p = 0.042) and positive (r_s_ = 0.147, p = 0.024) psychotic symptom scores, and with depressive (r_s_ = 0.165, p = 0.040) and manic symptom scores (r_s_ = 0.191, p = 0.008), while among HSV1+patients with BP with the negative symptom score (r_s_ = 0.243, p = 0.010). Finally, we reran the significant correlation analyses for (a) seronegative patients for CMV and TG, and (b) seropositive patients for either CMV or TG. Among SZ patients that were seronegative for both CMV and TG, there were no significant correlations between HSV1 IgG concentrations and total (r_s_ = 0.007, p = 0.956), general (r_s_ = 0.060, p = 0.641) or positive (r_s_ = −0.179, p = 0.167) psychotic symptom scores, or with manic symptom scores (r_s_ = 0.082, p = 0.557). By contrast, among SZ patients who were seropositive for CMV or TG, HSV1 IgG concentrations were significantly positively correlated with total (r_s_ = 0.218, p = 0.011), general (r_s_ = 0.189, p = 0.026) and positive (r_s_ = 0.248, p = 0.003) psychotic symptom scores, and with manic symptom scores (r_s_ = 0.202, p = 0.033). Concerning depressive symptoms, both patient groups showed similar non-significant positive correlations (r_s_ = 0.127, p = 0.390 and r_s_ = 0.104, p = 0.341, respectively). Finally, among BP patients that were seropositive for CMV or TG, there was no significant HSV1 antibody concentration-negative symptom score correlation (r_s_ = 0.195, p = 0.126), while among BP patients that were seronegative for CMV and TG, there was a significant positive correlation (r_s_ = 0.395, p = 0.034).

**Fig. 1:**
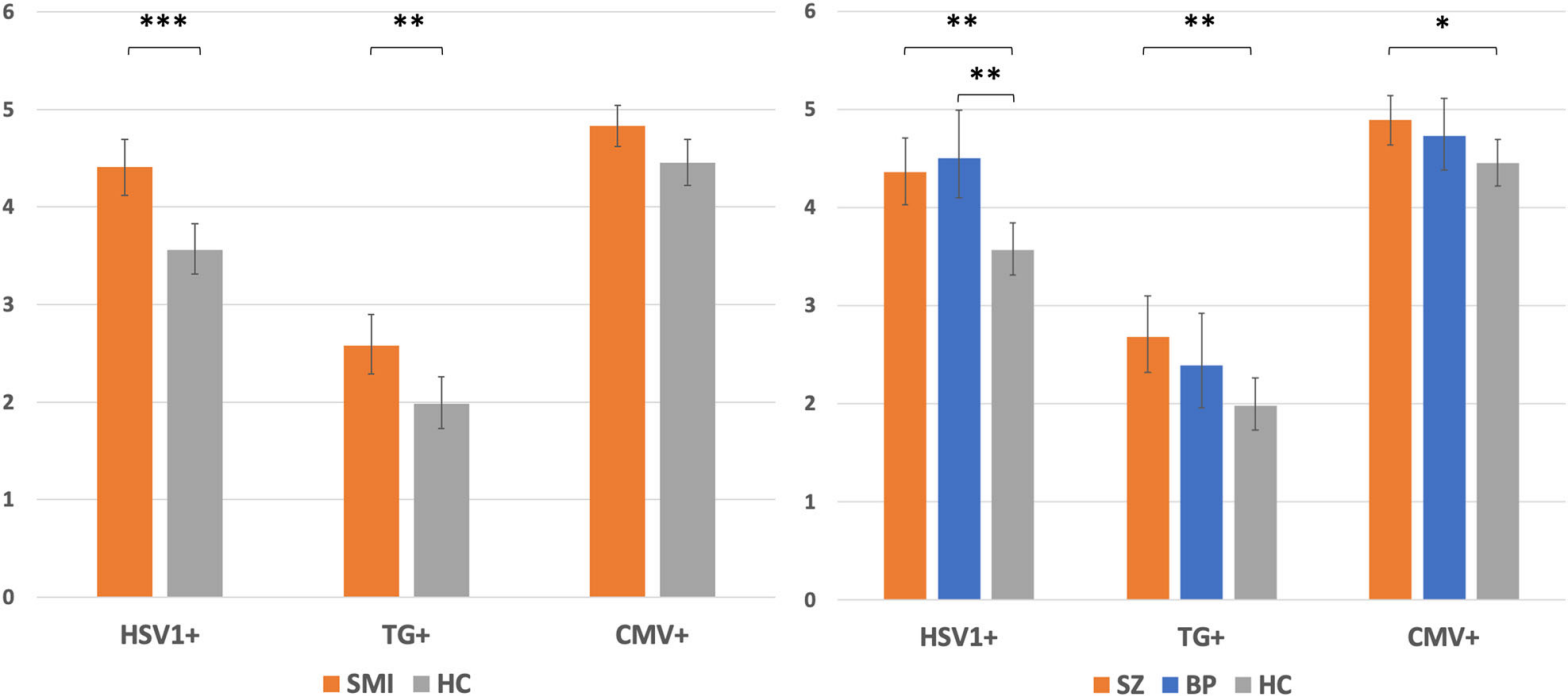
Back-transformed Herpes simplex virus 1 (HSV1), Cytomegalovirus (CMV) and Toxoplasma gondii (TG) immunoglobulin G (IgG) concentration means in (left) seropositive (HSV1+, TG+, and CMV+) patients with severe mental illness (SMI) and healthy controls (HC), and (right) seropositive patients with schizophrenia (SZ), bipolar disorder (BP) and HC. *<0.05, **<0.01, ***<0.001.

In our sex-, age- and education years-adjusted ANCOVA, the patient/control status was significantly associated with log_10_HSV1 concentrations, F(1554) = 17.978, p < 0.001, partial eta squared (η^2^) = 0.031, with higher log_10_HSV1 in patients than in HC (Fig. 1, left). As in the bivariate analysis, education years (p = 0.003, η^2^ = 0.016) were inversely associated with log_10_HSV1.

#### Toxoplasma gondii

In the bivariate analysis of TG seropositive (TG+) patients with SMI and TG+ HC (Table 3), there were no differences between patients and HC in sex distribution, age, or handedness. The SMI group had fewer education years, and higher AUDIT and DUDIT scores than HC (p < 0.001, 0.023, and <0.001, respectively). Among TG+ SMI patients as well as among TG+ HC, there were no correlations between TG IgG concentrations and IQ (r_s_ = −0.014, p = 0.881, and r_s_ = 0.059, p = 0.515, respectively).

**Table 3:** Group differences between Toxoplasma gondii (TG) immunoglobulin G (IgG) seropositive (TG+) patients with severe mental illness and healthy controls (HC) in sex, age, education years, handedness (right-handedness vs. left-handedness/ambidexterity), alcohol use disorder identification test (AUDIT) and drug use disorder identification test (DUDIT) scores.

	TG + patients		TG + HC		P-value^b^	Correlation with TG	
	N^a^	Mean (SD) or %	N^a^	Mean (SD) or %		±	P-value^c^
Sex (% women)	159	42.8	126	42.9	0.988	+	0.296
Age (years)	159	32.2 (11.7)	126	33.3 (8.8)	0.355	-	0.832
Education years	138	12.7 (2.3)	126	14.2 (2.2)	**<0.001**	-	0.060
Handedness (% right-handedness)	138	87.7	126	91.3	0.344	+	0.854
AUDIT score	116	7 (6.3)	92	5.5 (3.1)	**0.023**	+	0.787
DUDIT score	124	3.3 (6.8)	95	0.17 (0.63)	**<0.001**	+	0.778
DOI (years)	156	8.7 (7.7)				+	0.640
PANSS total score	156	54 (14.5)				+	0.068
YMRS score	127	3.7 (4)				+	0.097
IDS-C	108	16.6 (11.1)				+	0.993
On antipsychotics (%)	159	74.2				-	0.208
CPZ (mg/day)	115	320.6 (226.2)				+	0.209
On antidepressants (%)	159	32.7				+	0.167
On antiepileptics (%)	159	21.4				+	0.650
On lithium (%)	159	10.7				-	0.853

For patients, the duration of illness (DOI), Positive and Negative Syndrome Scale (PANSS) score, Young Mania Rating Scale (YMRS) score, Inventory of Depressive Symptoms, clinician rated, score (IDS-C), the percentage of patients on antipsychotics, antidepressants, antiepileptics, and lithium as well as the chlorpromazine equivalent doses (CPZ) among patients on antipsychotics are also shown. P values < 0.05 shown in bold.

^a^Number of participants with data in each variable.

^b^Chi-square test or t-test (Welch’s t-test in case of unequal variance).

^c^Point-biserial correlations for binary variables; Spearman’s correlations for quantitative variables.

In our sex- and age-adjusted ANCOVA, the patient/control status was significantly associated with log_10_TG IgG concentrations, F(1281) = 8.687, p = 0.003, η^2^ = 0.03, with higher log_10_TG in patients than in HC (Fig. 1, left).

#### Cytomegalovirus

In the bivariate analysis of CMV seropositive (CMV+) patients with SMI and CMV+ HC (Table 4), there were no differences between patients and HC in sex distribution, age, or handedness. Patients had fewer education years, and higher AUDIT and DUDIT scores than HC (p < 0.001 for all associations). Among CMV+ SMI patients as well as among CMV+ HC, there were no correlations between CMV IgG concentrations and IQ (r_s_ = −0.017, p = 0.751, and r_s_ = 0.062, p = 0.291, respectively).

**Table 4:** Group differences between Cytomegalovirus (CMV) immunoglobulin G (IgG) seropositive (TG+) patients with severe mental illness and healthy controls (HC) in sex, age, education years, handedness (right-handedness vs. left-handedness/ambidexterity), alcohol use disorder identification test (AUDIT) and drug use disorder identification test (DUDIT) scores.

	CMV + patients		CMV + HC		P-value^b^	Correlation with CMV	
	N^a^	Mean (SD) or %	N^a^	Mean (SD) or %		±	P-value^c^
Sex (% women)	447	49.4	296	46.6	0.452	+	0.118
Age (years)	447	32.9 (11.2)	296	33.8 (8.7)	0.221	+	0.167
Education years	404	12.7 (2.8)	296	14.4 (2)	**<0.001**	+	0.948
Handedness (% right-handedness)	403	90.8	296	90.2	0.783	-	0.696
AUDIT score	318	7.2 (7)	172	5.5 (3.5)	**<0.001**	-	0.170
DUDIT score	341	3.2 (7.7)	176	0.3 (1.5)	**<0.001**	+	0.642
DOI (years)	436	9.6 (9.2)				+	0.311
PANSS total score	435	57 (16.8)				-	0.955
YMRS score	378	4.82 (5)				+	0.227
IDS-C	315	18 (11.9)				+	0.764
On antipsychotics (%)	447	73.2				-	0.591
CPZ (mg/day)	322	316.6 (242.2)				+	0.612
On antidepressants (%)	447	33.1				-	0.118
On antiepileptics (%)	447	21.3				+	0.790
On lithium (%)	447	7.4				-	0.403

For patients, the duration of illness (DOI), Positive and Negative Syndrome Scale (PANSS) score, Young Mania Rating Scale (YMRS) score, Inventory of Depressive Symptoms, clinician rated, score (IDS-C), the percentage of patients on antipsychotics, antidepressants, antiepileptics, and lithium as well as the chlorpromazine equivalent doses (CPZ) among patients on antipsychotics are also shown. P values < 0.05 shown in bold.

^a^Number of participants with data in each variable.

^b^Chi-square test or t-test (Welch’s t-test in case of unequal variance).

^c^Point-biserial correlations for binary variables; Spearman’s correlations for quantitative variables.

In our sex- and age-adjusted ANCOVA, the patient/control status was nominally significantly associated with log_10_CMV IgG concentrations, F(1739) = 5.651, p = 0.018, η^2^ = 0.008, with higher log_10_CMV in patients than in HC (Fig. 1, left). After Bonferroni correction for multiple testing (alpha = 0.017) the association was no longer significant.

#### IL-18, hs-CRP, BAFF, and APRIL

HSV1, TG, and CMV IgG concentrations were all inversely correlated with IL-18 concentrations (r_s_ = −0.305, p < 0.001, r_s_ = −0.200, p < 0.001, and r_s_ = −0.142, p < 0.001, respectively). HSV1, but not TG or CMV, IgG concentrations were positively correlated with BAFF (r_s_ = 0.118, p = 0.004, r_s_ = 0.066, p = 0.271, and r_s_ = 0.035, p = 0.346, respectively). HSV1 and TG, but not CMV IgG concentrations were positively correlated with the BAFF/APRIL ratio (r_s_ = 0.271, p < 0.001, r_s_ = 0.204, p < 0.001, and r_s_ = 0.030, p = 0.422, respectively). HSV1 and TG, but not CMV, IgG concentrations were positively correlated with hs-CRP (r_s_ = 0.130, p = 0.002, r_s_ = 0.135, p = 0.027, and r_s_ = 0.004, p = 0.923, respectively).

### Post-hoc analysis

In the log_10_HSV1 ANCOVA, both SZ (p = 0.001) and BP (p = 0.001) had higher antibody levels than HC, whereas SZ and BP did not significantly differ. In the log_10_TG and log_10_CMV ANCOVAs, only SZ patients had higher antibody levels than HC (0.009 and 0.045, respectively), whereas BP and HC, and SZ and BP did not differ (Fig. 1, right). All four significant associations were confirmed with median regressions (p < 0.001, p < 0.001, p < 0.001, and p = 0.028, respectively).

## Discussion

In the present study investigating CMV, HSV1, and TG IgG positivity and concentrations in a large sample of SMI patients and HC, we found (i) significantly increased HSV1 and TG antibody concentration levels in seropositive SMI compared to seropositive HC groups and that (ii) higher HSV1 antibody levels among seropositive patients were associated with higher psychotic and depressive symptom scores. Interestingly, our post-hoc analysis revealed that both SZ and BP patients significantly differed in HSV1 antibody levels from HC, whereas for TG and CMV, SZ patients, but not BP patients, significantly differed from HC. In addition, in SZ, elevated HSV1 IgG concentrations correlated with higher scores of positive and general psychotic symptoms, as well as higher scores of manic and depressive symptoms, whereas in BP, higher HSV1 IgG levels were associated with higher scores of negative psychotic symptoms only. These findings collectively suggest that individuals with SZ experience a greater clinical burden related to neurotropic pathogens compared to those with BP. This observation aligns with our previous studies examining cognitive measures in relation to herpesviruses [13, 39]. Further, as hypothesized, the frequencies of CMV, HSV1, and TG seropositive individuals or the total pathogen load frequency (0–3) did not differ in patient groups relative to HC suggestive of a lack of an etiological role of CMV, HSV1, or TG in the development of SZ or BP. However, we did find a statistically non-significant 5% increase of CMV seropositivity in SZ relative to HC. Even though the lack of a significant CMV seropositivity-SZ association is in line with a recent meta-analysis [5], we cannot exclude that there is a true association; the need of even larger studies is thereby imperative.

Partially in line with the current CMV results, a large study of patients with SZ (n = 216), BP (n = 199), and HC (n = 80), showed that seropositive patients with SZ as well as seropositive patients with BP had higher CMV antibody concentrations than seropositive HC; however, no such associations were found for TG or HSV1 [40]. In another study of patients with SZ and HC, both CMV and HSV1 concentrations were higher in patients [41]. In a recent smaller study, patients with SZ (n = 28) did not differ from HC (n = 28), and neither did patients with BP (n = 32), in CMV, HSV1, or TG IgG concentrations [42]. Similarly, in another small study, there was no difference in CMV antibody levels between patients with SZ (n = 37) and HC (n = 16) [43]. Studying drug-naïve patients with recent-onset SZ (n = 38) and HC (n = 73), CMV, and TG, but not HSV1, antibody concentrations were higher among patients [44]. Finally, in a meta-analysis of TG in SZ, higher antibody levels were associated with the disorder [7] whereas in a meta-analysis of CMV concentration levels, there was no significant difference between patient with SZ and HC [5]. In the current study, we have included a substantially larger sample of patients and HC than in most previous original reports, and we also restricted the analysis to seropositive participants which may explain the discrepancy between results. In terms of studies of the potential role of infectious agents in psychiatric disorders, our study also strongly supports the analysis of quantitative levels of IgG antibodies as opposed to reliance on bivariate designations such as “positive” and negative”.

Previous reports have focused on the putative associations between antibody concentrations and both brain structural and clinical outcomes. For instance, investigating smaller samples overlapping with the current sample, CMV antibody concentrations were associated with smaller hippocampal dentate gyrus volume among men with SMI [45] as well as with smaller total surface area in patients with SZ [46], while HSV1 antibody concentrations were linked to smaller left caudal middle frontal, precentral, lingual, middle temporal and banks of superior temporal sulcus regional cortical gray matter volumes, among patients with SMI [39]. Further, elevated HSV1 antibody concentrations have been associated with poorer cognitive performance in SZ [32]. To the best of our knowledge, there are no previous reports implicating antibody concentration levels in psychotic symptom severity among HSV1-exposed patients with SMI. We have here shown that among HSV1-exposed patients, HSV1 antibody levels were associated with more severe psychosis shown as higher PANSS total score; follow-up analyses showed associations with both positive, negative, and general symptom scores. These are novel findings suggesting that HSV1-exposed patients exhibit a heightened immune response, possibly reflecting an increased infection severity, that appears to be associated with the severity of their psychotic symptoms. The results were in the same direction for TG but did not reach the significance threshold (PANSS total score; p = 0.068). This could possibly be due to the smaller sample size in the TG analysis as TG had substantially lower seroprevalence than HSV1. Finally, we have previously shown that both HSV1- and CMV-exposed SMI patients have brain structure aberrations [39, 46], but for HSV1 only, these aberrations were restricted to patient groups, suggesting that HSV1 may promote a more severe infection in SMI relative to HC in line with the current results.

Interestingly, our study demonstrated significant positive correlations between HSV1 IgG concentrations and symptom scores—including total, general, positive psychotic, as well as manic symptom scores—in the subgroup of SZ patients also infected with CMV or TG. These correlations were notably stronger than those observed in the overall patient cohort. Conversely, in the absence of concurrent CMV and TG infections, no significant correlations were found between HSV1 IgG concentrations and symptom scores. Consequently, our results indicate that in SZ patients, elevated HSV1 antibody concentrations are associated with increased symptomatology primarily, or even exclusively, in the presence of concurrent CMV or TG infections. Based on these findings, the relationship between HSV1, CMV, and TG appears to be synergistic in SZ. In BP patients, the correlation between HSV1 IgG concentrations and negative symptom scores was only significant in the absence of CMV and TG infections. We speculate that the diminished effect of HSV1 in the presence of CMV or TG in BP patients may suggest an antagonistic relationship, where the co-infections might reduce the influence of HSV1 on symptomatology. However, the lack of any correlation between HSV1 IgG concentrations and manic symptoms, the core symptoms of BP, diminishes the likelihood that HSV1 infection has a clinically meaningful impact on the overall symptomatology of BP.

The higher antibody levels observed in individuals with SZ or BP compared to HC suggests a heightened immune response against the studied pathogens. This heightened immune response may reflect a more serious infection in patients with SMI, alternatively an altered immune response unrelated to the severity of the infection. In this context, there is considerable evidence of B-cell dysfunction in SMI [18]. B cells have various functions including the production of antibodies and cytokines, and both elevated antibody and cytokine production have been shown in SZ [18]. We found associations between higher antibody levels and lower IL-18 levels for all three pathogens, which was unexpected. Additionally, we found higher BAFF levels associated with HSV1, as well as a higher BAFF/APRIL ratio and elevated hs-CRP levels for both HSV1 and TG. IL-18 is a critical cytokine involved in pro-inflammatory responses against infections [21], and its attenuated levels in association with higher antibody levels may suggest a regulatory mechanism to mitigate excessive inflammation and tissue damage in latently infected individuals. This downregulation in relation to increased antibody levels may also be indicative of T cell exhaustion, where continuous exposure to antigens leads to a diminished immune response over time [47]. In particular, as exhausted T cells may produce fewer pro-inflammatory cytokines, and IL-18 has been implicated in T cell exhaustion [48], this phenomenon could contribute to the observed decrease in IL-18 levels. On the other hand, the positive associations between HSV1 and TG antibody levels and hs-CRP suggest that HSV-1 and TG infections may still drive systemic inflammation, likely through pathways independent of IL-18. Concurrently, the elevated BAFF levels, reflecting increased B-cell responses [19], support the notion of a hypothesized heightened immune response and constitutes a strategic immune adjustment to sustain control over the persistent pathogen. Finally, the increased BAFF/APRIL ratio for both HSV1 and TG might indicate an augmented central nervous system pathology with increasing antibody levels [20]; this finding aligns with the observed associations of antibody levels with increased symptom scores described in the previous paragraph.

Lower socioeconomic status has been linked to immune system aberrations [49]. In the present study, we used years of education as a proxy for socioeconomic status [24]. A previous report demonstrated an inverse association between years of education, as well as other markers of lower socioeconomic status, and CMV IgG titers [50]. We found that among HSV1+ (but not CMV+ or TG+) participants as whole (i.e., patients and HC), fewer years of education correlated with increased HSV1 IgG concentrations. This association may be attributable to socioeconomic status-related lifestyle behaviors and psychosocial stressors that could modulate immune function and IgG production. As HSV1+ patients and HSV1+ HC significantly differed in years of education (Table 1), we adjusted our main model for education years and found that there was still a significant difference in IgG levels between SMI patients and HC. This suggests that the higher HSV1 IgG concentrations observed in SMI patients compared to HC cannot be explained solely by differences in socioeconomic status between the two groups.

Studying an overlapping sample, we have previously investigated putative associations between CMV and HSV1 seropositivity or antibody concentrations and both cognitive [13, 39] and brain MRI measures in SMI and HC [39, 45, 46]. In sum, our previous results suggested that CMV and HSV1 seropositivity are linked to cognitive and brain structure aberrations largely restricted to SMI patient groups. Our current results on IQ indicate that among seropositive patients, HSV1 and CMV antibody concentrations were not associated with cognitive aberrations, diminishing the likelihood of a dose-response association between HSV1 or CMV antibody levels and IQ. Regarding TG, we have reported that TG seropositivity is associated with increased circulatory neuron-specific enolase, a marker of brain damage, and IL-18, among both patients with SMI and HC [51]. We have not previously investigated antibody concentration differences between exposed SMI patients and HC for any pathogen, which is the main analysis of the present study. Regarding pathogen seropositivity, we have previously reported a lack of association between CMV and SMI/HC status [13], but not for HSV1, TG, or the pathogen load.

The present results could fill knowledge gaps regarding the pathophysiology of SMI, and most importantly may have short- and long-term benefits for patients with SMI and individuals at high-risk of SMI. New-generation antiviral and antiprotozoal medications are already available. There are currently no approved vaccines, although there is ongoing research with promising results [52]. This paradigm shift could result in the initiation of large-scale and extended clinical trials which are necessary to determine whether medication can indeed ameliorate the symptoms of patients with SZ or BP. Further, the current report combined with previous research could lead to a prioritization of patients with SMI or individuals at high-risk of SMI once vaccines become available.

The study has certain limitations. First, there are several biological explanations for the higher antibody concentrations in the patient groups. They may not reflect an enhanced infection severity accompanied with a heightened immune response, but also a more recent infection or even immune system abnormalities linked to SMI and unrelated to infection severity or timing [18, 33, 53]. In addition, we cannot exclude the possibility of reverse causality, wherein psychotic symptoms may induce significant chronic stress and activation of the hypothalamic-pituitary-adrenal axis. This activation, in turn, can modulate immune function by suppressing certain aspects of the immune response while enhancing others, including increasing antibody levels [54]. Furthermore, this is not a prospective study, and therefore, we cannot determine when the seropositive patients were exposed to the studied pathogens. As we cannot ascertain if the exposure preceded the development of SMI, we cannot rule out that the lack of associations between HSV1, CMV, or TG seropositivity and SMI/HC status may be due to the cross-sectional design of our study, which does not account for the temporality of events. Interestingly, in a large-scale study on TG and CMV, the associations between TG and SZ, as well as between CMV and any psychiatric disorder, became stronger when the authors accounted for temporality [4]. However, for the main hypotheses of our study, specifically the differences in antibody levels between infected patients and HC, as well as the associations between antibody levels and symptom scores and immune marker levels, we consider the cross-sectional design to be appropriate. Further, we have data available on current medication but limited data on lifetime medication. Consequently, we are unfortunately unable to reliably study new-onset drug-naïve patients. Finally, all psychosis symptom scores were moderately to strongly (and highly significantly) correlated with depression symptom scores (Supplementary Table 1), and as there is compelling evidence between inflammation and depression [55, 56], we cannot exclude that the observed HSV1-psychotic symptoms associations reflect a primary HSV1-depressive symptoms association.

To conclude, while SMI patients did not have increased rates of seropositivity to common infectious agents such as CMV, HSV1, or TG, or an increased pathogen load compared to HC, exposed SMI patients had higher HSV1 (both for SZ and BP), TG and CMV (for SZ) antibody concentrations as compared to exposed HC. This may be attributed to either a more severe infection causing a heightened immune response, or to a primary immune system hyperactivity in SMI that is independent of infection severity. The latter implies that the suggested mild inflammatory state in SMI could be an epiphenomenon of a dysregulated immune function. Furthermore, higher HSV1 antibody levels among HSV1-exposed SZ patients were associated with higher psychotic symptom scores, especially in the case of coinfection with CMV or TG, suggesting a synergistic effect. Among all participants, HSV1 IgG concentrations were inversely associated with IL-18 and positively associated with BAFF and hs-CRP, possibly indicating T cell exhaustion, increased B-cell response, and enhanced inflammation, respectively. Additional studies should be directed at uncovering the mechanisms underlying the increased levels of IgG antibodies in individuals with psychiatric disorders with the goal of identifying pathways which might be amenable to the development of biomarkers of novel interventions.

## Supplementary information

Suppl. Material

## Data Availability

Data supporting the findings of the present study have repository at NORMENT/Oslo University Hospital. Restrictions apply to the availability of data and they are thereby not publicly available. Data can be made available under reasonable request and with permission of NORMENT/Oslo University Hospital, in accordance with the ethics agreements/research participants consent.
