# Supplementary material for: Increased Herpes simplex virus 1, Toxoplasma gondii and Cytomegalovirus antibody concentrations in severe mental illness
Source: Transl Psychiatry. 2024 Dec 18;14:498. doi: 10.1038/s41398-024-03198-y (PMC11655861; doi:10.1038/s41398-024-03198-y)
Supplement: Supplementary file 1 — Suppl. Material [file 41398_2024_3198_MOESM1_ESM.pdf]

Supplementary material

Power analysis

In our previous studies on CMV and HSV1 on brain measures and general intelligence, the effect sizes were small to medium (partial eta^2 0.03-0.06) [1-3]. In the present study, the sample size to ensure a power of 95% to detect small effect sizes was 423 (partial eta^2=0.03, effect size f=0.1758631, a err prob=0.05, power=0.95), while the sample size to ensure a power of 95% to detect medium effect sizes was 206 (partial eta^2=0.06, effect size f=0.2526456, a err prob=0.05, power=0.95) [4]. We included 743 CMV+ participants, 593 HSV1+ participants and 285 TG+ participants, ensuring adequate (>95%) power for medium effects for all three groups, and for small effects for the CMV+ and HSV1+ groups. The power to detect small effects for the TG+ participants analysis was 84%. Of note, the TG+ participants analysis provided a significant result (p=0.003).

HSV1

In the ANCOVA on $\log_{10}$HSV1, the back-transformed adjusted marginal HSV1 means were 4.41 (95% CI, 4.13 to 4.69) and 3.56 (95% CI, 3.31 to 3.83) for patients and HC, respectively (Figure 1). There was homogeneity of variances evaluated with Levene's test, p=0.108. The residuals of the overall model were approximately normally distributed determined by visual inspection. Observations with studentized residuals greater than 3 in absolute value were considered outliers. There were no outliers. We ran sensitivity analysis applying a median regression on HSV1 IgG concentrations. As in the ANCOVA on $\log_{10}$TG, patients had significantly higher levels than HC (p<0.001).

TG

In the ANCOVA on $\log_{10}$TG, the back-transformed adjusted marginal TG means were 2.58 (95% CI, 2.29 to 2.9) and 1.98 (95% CI, 1.73 to 2.26) for patients and HC, respectively (Figure 1). There was homogeneity of variances evaluated with Levene's test, p=0.710. The residuals of the overall model were not normally distributed determined by visual inspection. Observations with studentized residuals greater than 3 in absolute value were considered outliers. There were no outliers. Due to the non-normal distribution of the residuals, we ran sensitivity analysis applying a median regression on TG IgG concentrations. As in the ANCOVA on $\log_{10}$TG, patients had significantly higher levels than HC (p<0.001).

CMV

In the ANCOVA on $\log_{10}$CMV, the back-transformed adjusted marginal CMV means were 4.83 (95% CI, 4.62 to 5.04) and 4.45 (95% CI, 4.22 to 4.69) for patients and HC, respectively (Figure 1). There was a deviation from homogeneity of variances evaluated with Levene's test, p<0.001. The residuals of the

overall model were approximately normally distributed determined by visual inspection. Observations with studentized residuals greater than 3 in absolute value were considered outliers. There were 17 outliers. These cases did not exhibit high leverage (all up to 0.02) or high Cook's distance value (0.01-0.07) and were not excluded. Due to the deviation from the homogeneity of variances assumption, we ran sensitivity analysis applying a median regression on CMV IgG concentrations. Patients had non-significantly higher levels of CMV IgG levels than HC (p=0.086).

Age-, sex-, education- and AUDIT-adjusted ANCOVAs

Applying age-, sex-, education years- and AUDIT-adjusted ANCOVAs, we ran sensitivity analyses for all three pathogens. The patient/control status was significantly associated with $\log_{10}$HSV1 concentrations, $F(1,363)=57.413$, $p<0.001$, with higher $\log_{10}$HSV1 in SMI patients than in HC. The patient/control status was significantly associated with $\log_{10}$TG concentrations, $F(1,188)=5.861$, $p=0.016$, with higher $\log_{10}$TG in SMI patients than in HC. The patient/control status was not significantly associated with $\log_{10}$CMV concentrations, $F(1,452)=2.516$, $p=0.113$, with non-significantly higher $\log_{10}$CMV in SMI patients than in HC.

Valproate and Toxoplasma gondii

It has been reported that among antipsychotics and mood stabilizers, sodium valproate and haloperidol show the strongest in vitro inhibition of TG whereas other antipsychotics, including risperidone, clozapine, olanzapine and quetiapine, as well as lithium have much weaker or no anti-TG activity [5]. We had 39 TG seronegative SMI patients currently on valproate and 12 TG seropositive SMI patients currently on valproate. We conducted an explanatory analysis investigating A) The association between TG seropositivity and current valproate use (yes/no) among SMI patients. There was no association assessed with chi-square test (p=0.617). B) The association between current valproate use (yes/no) and TG IgG concentrations. There was no association assessed with t test (p=0.727). C) The correlations between valproate dose and serum concentrations with TG IgG concentrations. There were no correlations between valproate daily dose ($r_s=0.270$, $p=0.396$) or serum concentrations ($r_s=0.178$, $p=0.601$) and IgG TG concentrations assessed with Spearman’s correlations. There were no TG seropositive SMI patients who used haloperidol.

	IDS-C	
	**R_s**	**P-values**
**PANSS total**	0.500	<0.001
**Positive**	0.319	<0.001
**Negative**	0.340	<0.001
**General**	0.580	<0.001

Supp. Table 1. Spearman's correlations between Positive and Negative Syndrome Scale (PANSS) scores, and Inventory of Depressive Symptoms, clinician rated, score (IDS-C) among Herpes Simplex Virus 1 seropositive patients with severe mental illness

References

1. Andreou, D., Jorgensen, K. N., Wortinger, L. A., Engen, K., Vaskinn, A., Ueland, T. *et al.* Cytomegalovirus infection and IQ in patients with severe mental illness and healthy individuals. *Psychiatry Res* **300**, 113929, doi:10.1016/j.psychres.2021.113929 (2021).
2. Andreou, D., Jorgensen, K. N., Nerland, S., Engen, K., Yolken, R. H., Andreassen, O. A. *et al.* Cytomegalovirus infection associated with smaller dentate gyrus in men with severe mental illness. *Brain Behav Immun* **96**, 54-62, doi:10.1016/j.bbi.2021.05.009 (2021).
3. Andreou, D., Jorgensen, K. N., Nerland, S., Ueland, T., Vaskinn, A., Haukvik, U. K. *et al.* Herpes simplex virus 1 infection on grey matter and general intelligence in severe mental illness. *Transl Psychiatry* **12**, 276, doi:10.1038/s41398-022-02044-3 (2022).
4. Faul, F., Erdfelder, E., Lang, A. G. & Buchner, A. G*Power 3: a flexible statistical power analysis program for the social, behavioral, and biomedical sciences. *Behav Res Methods* **39**, 175-191, doi:10.3758/bf03193146 (2007).
5. Jones-Brando, L., Torrey, E. F. & Yolken, R. Drugs used in the treatment of schizophrenia and bipolar disorder inhibit the replication of Toxoplasma gondii. *Schizophr Res* **62**, 237-244, doi:10.1016/s0920-9964(02)00357-2 (2003).
